# Aktuelle Zahlen zur rheumatologischen Versorgung – Jahresbericht aus der Kerndokumentation der regionalen kooperativen Rheumazentren

**DOI:** 10.1007/s00393-024-01497-9

**Published:** 2024-04-03

**Authors:** Katinka Albrecht, Katja Thiele, Tobias Alexander, Martin Aringer, Thorsten Eidner, Jörg Henes, Guido Hoese, Kirsten Karberg, Uta Kiltz, Andreas Krause, Wolfgang Ochs, Jutta G Richter, Susanna Späthling-Mestekemper, Mirko Steinmüller, Siegfried Wassenberg, Anja Strangfeld, Johanna Callhoff

**Affiliations:** 1 Programmbereich Epidemiologie und Versorgungsforschung, Deutsches Rheuma-Forschungszentrum Berlin, Charitéplatz 1, 10117 Berlin, Deutschland; 2 Medizinische Klinik m.S. Rheumatologie und Klinische Immunologie, Charité – Universitätsmedizin Berlin, corporate member Freie Universität Berlin, Humboldt-Universität zu Berlin, und Berlin Institute of Health (BIH), Berlin, Deutschland; 3 Rheumatologie, Medizinische Klinik und Poliklinik III, Universitätsklinikum und Medizinische Fakultät Carl Gustav Carus, Technische Universität Dresden, Dresden, Deutschland; 4 Klinik für Innere Medizin III – Rheumatologie/Osteologie, Universitätsklinikum Jena, Jena, Deutschland; 5 Zentrum für Interdisziplinäre Rheumatologie, klinische Immunologie und Autoimmunerkrankungen (INDIRA), Department für Innere Medizin II, Universitätsklinik Tübingen, Tübingen, Deutschland; 6 Rheumapraxis Stadthagen, Stadthagen, Deutschland; 7 Rheumatologisches Versorgungszentrum Berlin-Steglitz, Berlin, Deutschland; 8 Ruhr-Universität Bochum, Bochum, Deutschland; 9 Rheumazentrum Ruhrgebiet, Herne, Deutschland; 10 Rheumatologie, klinische Immunologie und Osteologie, Immanuel-Krankenhaus Berlin, Berlin, Deutschland; 11 Internistisch-rheumatologische Gemeinschaftspraxis, Bayreuth, Deutschland; 12 Klinik für Rheumatologie, Medizinische Fakultät, Universitätsklinikum Düsseldorf, Heinrich-Heine-Universität Düsseldorf, Düsseldorf, Deutschland; 13 Hiller Forschungszentrum Rheumatologie, Medizinische Fakultät, Universitätsklinikum Düsseldorf, Heinrich-Heine-Universität Düsseldorf, Düsseldorf, Deutschland; 14 Rheumapraxis München-Pasing, München, Deutschland; 15 Rheumapraxis Dilltal, Ehringshausen, Burbach, Ehringshausen, Deutschland; 16 Rheumazentrum Ratingen, Rheumatologische Gemeinschaftspraxis, Ratingen, Deutschland; 17 Medizinische Klinik mit Schwerpunkt Rheumatologie und Klinische Immunologie, Charité-Universitätsmedizin Berlin, Berlin, Deutschland; 18 Institut für Sozialmedizin, Epidemiologie und Gesundheitsökonomie, Charité-Universitätsmedizin Berlin, Berlin, Deutschland

**Keywords:** Versorgungsforschung, Rheumatologie, Rheumatische Erkrankungen, Rheumatoide Arthritis, Langzeitbeobachtung, Health care research, Rheumatology, Rheumatic diseases, Rheumatoid arthritis, Long-term observational research

## Abstract

Die Kerndokumentation der regionalen kooperativen Rheumazentren erfasst seit 1993 jährlich Daten zur rheumatologischen Versorgung von Patient:innen mit entzündlich-rheumatischen Erkrankungen. In diesem erstmals erscheinenden Jahresbericht werden aktuelle Querschnittsdaten zur Medikation und zu patientenberichteten Outcomes vorgestellt, die im Jahr 2022 erhoben wurden.

## Abstract (en)

In the National database (NDB) of the German regional collaborative arthritis centres, annual data on the rheumatological care of patients with inflammatory rheumatic diseases have been collected since 1993. This first annual report presents current cross-sectional data on medication and patient-reported outcomes gathered in 2022.

## Einleitung

In der Kerndokumentation der regionalen kooperativen Rheumazentren werden jährlich wesentliche Daten zur rheumatologischen Versorgung von Patient:innen mit entzündlich-rheumatischen Erkrankungen und zu ihrer Krankheitslast erhoben. Ergebnisse und Versorgungstrends werden jedes Jahr anhand von Ergebnisgrafiken gezeigt [1]. Auf vielfachen Wunsch veröffentlichen wir dieses Jahr erstmalig zusätzlich einen Jahresbericht, um die aktuellsten Daten auch in der *Zeitschrift für Rheumatologie* sichtbar zu machen. Da wir ausschließlich Querschnittsdaten berichten, sind alle Werte als Momentaufnahmen im kontinuierlichen Behandlungsprozess zu bewerten.

## Methodik

Die Kerndokumentation ist eine seit 1993 laufende bundesweite prospektive Langzeitdokumentation. Konsekutive Patient:innen aus der rheumatologischen Routineversorgung werden einmal im Jahr hinsichtlich ihres Krankheitsverlaufes dokumentiert [2]. Rheumatolog:innen dokumentieren u. a. die Krankheitsaktivität und die aktuelle Medikation. Die Krankheitsaktivität wird bei allen Krankheitsentitäten mit einer numerischen Ratingskala (NRS) von 0 bis 10 erfasst, wobei 0 keiner Aktivität entspricht. Für die rheumatoide Arthritis (RA) wird zusätzlich der Disease Activity Score (DAS28) bzw. der Clinical oder Simple Disease Activity Index (CDAI oder SDAI) erhoben, für die axiale Spondyloarthritis (axSpA) der Bath Ankylosing Spondylitis Disease Activity Index (BASDAI) und der Axial Spondyloarthritis Disease Activity Score (ASDAS). Beim systemischen Lupus erythematodes (SLE) wird in einigen Einrichtungen der European Consensus Lupus Activity Measurement (ECLAM) erhoben.

An medikamentösen Therapien werden Glukokortikoide (GC), alle konventionell synthetischen (cs), biologischen (b) und zielgerichteten (ts) immunmodulierenden Medikamente (englisch disease-modifying antirheumatic drugs, DMARDs), nichtsteroidale Antirheumatika (NSAR), nicht-opioidhaltige Analgetika und Opioide erfasst. Die Abfrage nichtmedikamentöser Therapien umfasst Physiotherapie, Ergotherapie, Rheumafunktionstraining und Patientenschulung.

Patientenberichtete Outcomes (PRO) beinhalten u. a. den allgemeinen Gesundheitszustand, Krankheitsaktivität, Schmerzen, Erschöpfung/Müdigkeit, Schlafstörungen, Schwierigkeiten bei körperlichen Tätigkeiten sowie psychisches und körperliches Wohlbefinden, jeweils bezogen auf die letzte Woche. Diese werden auch mit einer NRS, adaptiert vom Rheumatoid Arthritis Impact of Disease (RAID) [3] erfasst. Für die Funktionsfähigkeit wird bei RA der Funktionsfragebogen Hannover (FFbH) und bei axSpA der Bath Ankylosing Spondylitis Functional Index (BASFI) erhoben.

Einschlusskriterien: In die Kerndokumentation können alle Patient:innen mit entzündlich-rheumatischen Erkrankungen eingeschlossen werden. Die Daten aus diesem Bericht beziehen sich auf das Auswertungsjahr 2022. Für die Darstellung von Patientencharakteristika, Therapien und PROs sind nur Patient:innen mit einer gesicherten Diagnose berücksichtigt.

## Ergebnisse

Im Jahr 2022 erhoben 13 rheumatologische Einrichtungen (6 Praxen, 1 Krankenhaus und 6 Universitätskliniken) Daten für die Kerndokumentation. Dokumentiert wurden insgesamt 13.887 ambulante Patient:innen. Davon hatten 47 % Arthritiden, 26 % Spondyloarthritiden, 15 % Kollagenosen, 7 % Vaskulitiden, 4 % eine Polymyalgia rheumatica (PMR) und 1 % eine sonstige Diagnose. Die häufigsten Krankheitsentitäten waren RA (*n* = 5998), Psoriasis-Arthritis (PsA: *n* = 1795), axSpA (*n* = 1449) und SLE (*n* = 946), s. Tab. 1. Knapp die Hälfte der Patient:innen (48 %) wurde über die ambulante spezialfachärztliche Versorgung (ASV), 39 % über die Regelversorgung und 5 % über eine Hochschul‑/Ermächtigungsambulanz betreut.

**Tab. 1:** Patientencharakteristika, Daten aus der Kerndokumentation von 2022

	RA	PsA	axSpA	SLE	SSc	SjS	IIM	MCTD	PMR	RZA	AAV	BD
*N*	5998	1795	1449	946	367	253	106	144	486	355	332	192
Gesicherte Diagnose, *N*	5791	1715	1376	890	344	236	97	137	452	339	305	154
Weiblich (%)	73	54	37	87	76	89	64	85	61	74	55	46
BMI, MW (SD)	27 (6)	29 (6)	27 (5)	26 (6)	25 (6)	25 (7)	27 (6)	26 (6)	26 (4)	26 (5)	26 (5)	26 (4)
BMI ≥ 30 kg/m^2^ (%)	24	33	27	20	13	15	17	23	18	16	21	14
Alter in 2022 in Jahren, MW (SD)	63 (14)	56 (14)	49 (14)	49 (15)	58 (14)	56 (14)	56 (16)	52 (15)	72 (9)	73 (9)	60 (14)	45 (11)
Erkrankungsalter in Jahren, MW (SD)	49 (16)	43 (15)	31 (12)	32 (14)	45 (14)	43 (15)	46 (16)	37 (15)	67 (3)	67 (4)	49 (9)	28 (14)
Krankheitsdauer in 2022 in Jahren, MW (SD)	14 (11)	14 (11)	19 (13)	18 (11)	12 (9)	12 (9)	10 (8)	15 (10)	5 (6)	6 (5)	11 (9)	16 (10)
Krankheitsdauer, Median	11	11	16	17	11	11	9	15	3	4	9	14
Krankheitsdauer < 2 Jahre (%)	9	9	5	5	8	8	16	9	38	23	12	1

*RA* rheumatoide Arthritis, *PsA* Psoriasis-Arthritis, *axSpA* axiale Spondyloarthritis, *SLE* systemischer Lupus erythematodes, *SSc* systemische Sklerose, *SjS* primäres Sjögren-Syndrom, *IIM* idiopathische inflammatorische Myositiden, *MCTD* mixed connective tissue diseases, *PMR* Polymyalgia rheumatica, *RZA* Riesenzellarteriitis, *AAV* ANCA-assoziierte Vaskulitiden, *BD* Morbus Behçet, *MW* Mittelwert, *SD* Standardabweichung, *BMI* Body-Mass-Index

### Patientencharakteristika

Das mittlere Alter der Patient:innen variierte von 45 Jahren bei Behçet-Erkrankung (BD) bis 73 Jahre bei Riesenzellarteriitis (RZA). Die Krankheitsdauer lag im Median zwischen 3 Jahren bei PMR und 16 Jahren bei axSpA. Das mittlere Alter bei Erkrankung lag zwischen 28 Jahren bei BD und 67 Jahren bei PMR und RZA. Ein Prozent (BD) bis 38 % (PMR) hatten eine Krankheitsdauer unter zwei Jahren. Der Anteil an Frauen war mit 89 % bei primärem Sjögren-Syndrom (SjS) am höchsten und mit 37 % bei axSpA am niedrigsten.

### Krankheitsaktivität und Remission

Die ärztlich dokumentierte Krankheitsaktivität nach der NRS lag krankheitsübergreifend im niedrigen Bereich: Der Mittelwert nach der NRS war am niedrigsten bei BD (0,9 ± 1,2) und am höchsten bei SSc (2,4 ± 1,5). Bei 3 % (BD) bis 19 % (SSc) stuften die Rheumatolog:innen die Aktivität als moderat bis hoch (> 4) ein, s. Tab. 2. Bei RA lag die Einschätzung bei 35 % der Betroffenen bei 0, bei 27 % bei 1 und bei 15 % bei 2 (insgesamt 77 %: 0–2).

**Tab. 2:** Krankheitsaktivität nach ärztlicher Einschätzung auf einer numerischen Ratingskala von 0–10

	RA	PsA	axSpA	SLE	SSc	SjS	IIM	MCTD	PMR	RZA	AAV	BD
Krankheitsaktivität, ärztliche Einschätzung, NRS 0–10, MW (SD)	1,6 (1,8)	1,7 (1,8)	1,7 (1,8)	1,6 (1,5)	2,4 (1,5)	1,7 (1,6)	1,9 (1,6)	1,7 (1,5)	1,0 (1,5)	1,2 (1,5)	1,4 (1,4)	0,9 (1,2)
Niedrig (0–3), %	86	85	85	90	81	87	87	92	92	92	92	97
Moderat bis hoch (4–10), %	14	15	15	10	19	13	13	8	8	8	8	3

*RA* rheumatoide Arthritis, *PsA* Psoriasis-Arthritis, *axSpA* axiale Spondyloarthritis, *SLE* systemischer Lupus erythematodes, *SSc* systemische Sklerose, *SjS* primäres Sjögren-Syndrom, *IIM* idiopathische inflammatorische Myositiden, *MCTD* mixed connective tissue diseases, *PMR* Polymyalgia rheumatica, *RZA* Riesenzellarteriitis, *AAV* ANCA-assoziierte Vaskulitiden, *BD* Morbus Behçet, *MW* Mittelwert, *SD* Standardabweichung, *BMI* Body-Mass-Index, *NRS* numerische Ratingskala, 0 entspricht inaktiver und 10 hoch aktiver Erkrankung

Von den RA-Patient:innen befanden sich 46 % in DAS28-Remission (< 2,6), 19 % hatten eine niedrige (2,6–3,2), 31 % eine mittlere (> 3,2–5,1) und 4 % (> 5,1) eine hohe Krankheitsaktivität. Nach dem CDAI waren 24 % in Remission (≤ 2,8), 51 % hatten eine niedrige (2,8–10), 20 % eine moderate (11–22) und 5 % eine hohe (> 22) Krankheitsaktivität.

**Abb. 1:**
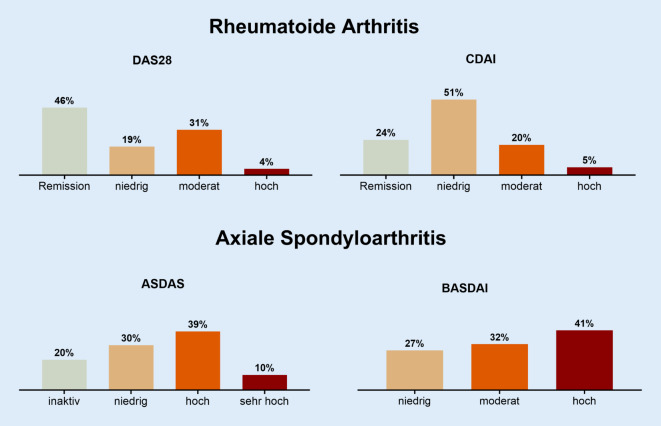
Remission und Krankheitsaktivität bei rheumatoider Arthritis und axialer Spondyloarthritis. Dargestellt sind die prozentualen Anteile an Patient:innen aus der Kerndokumentation mit rheumatoider Arthritis bzw. axialer Spondyloarthritis, die sich in Remission befinden oder eine niedrige/moderate/hohe Krankheitsaktivität haben. *DAS28* Disease Activity Score, *CDAI* Clinical Disease Activity Score, *ASDAS* Axial Spondyloarthritis Disease Activity Score, *BASDAI* Bath Ankylosing Spondylitis Disease Activity Score

Von den axSpA-Patient:innen hatten 20 % gemäß dem ASDAS-CRP eine inaktive Erkrankung (< 1,3) und 30 % eine niedrige Krankheitsaktivität. Nach dem BASDAI hatten 27 % eine niedrige (0–2), 32 % eine mittlere (< 2–4) und 41 % eine hohe Krankheitsaktivität (> 4), s. Abb. 1.

Der ECLAM wurde bei 141 SLE-Patient:innen erfasst. Nach dem ECLAM Score (0–10, 0 entsprechend einer inaktiven Erkrankung) betrug der Score bei 4 % null, bei 43 % eins, bei 45 % zwei, bei 6 % drei und bei 2 % vier. Am häufigsten waren eine hämatologische Beteiligung (48 %) und Komplementerniedrigung (29 %).

### Glukokortikoide, NSAR und Schmerzmedikation

In 2022 erhielten bei RA 28 % Glukokortikoide (GC), bei SLE 46 % und bei ANCA-assoziierten Vaskulitiden (AAV) 62 %, die Prozentzahlen für die weiteren Diagnosen sind in Tab. 2 angeführt. Davon hatten etwa 80 % eine niedrige Dosis von bis zu 5 mg Prednisolonäquivalent pro Tag. Nichtsteroidale Antirheumatika wurden vor allem bei axSpA (60 %), PsA (41 %) und RA (40 %) eingesetzt. Zwölf bis 20 % der Patient:innen erhielten nicht-opioidhaltige Analgetika und 2 % (BD) bis 9 % (MTCD) nahmen aktuell ein Opioid ein (s. Tab. 3).

**Tab. 3:** Konventionelle medikamentöse Therapien

	RA	PsA	axSpA	SLE	SSc	SjS	IIM	MCTD	PMR	RZA	AAV	BD
*N mit Therapieangabe*	*5791*	*1715*	*1376*	*890*	*344*	*236*	*97*	*137*	*452*	*339*	*305*	*154*
Glukokortikoide	28	11	5,7	46	18	27	46	40	59	49	62	40
– Davon ≤ 5 mg/d	79	71	62	78	80	84	71	80	71	69	82	90
NSAR	40	41	60	23	16	26	14	23	24	14	12	16
Nicht-opioidhaltige Analgetika	17	13	15	17	10	19	13	16	18	20	16	12
Opioide	6,6	7,3	6,9	4,8	3,2	6,5	5,2	9,5	5,3	5,6	5,3	2,0
Basistherapie (cs/b/ts DMARD)	89	85	70	89	63	65	77	81	32	64	81	60
csDMARD	67	46	11	86	53	61	72	76	31	34	58	37
Azathioprin	0,3	0,1	0,3	17	5,6	5,5	13	10	0,7	2,4	25	25
Ciclosporin A	–	0,3	–	1,7	–	–	3,1	–	–	–	–	1,9
Cyclophosphamid	0,1	–	0,1	0,4	0,6	–	–	–	–	–	1,0	–
Hydroxychloroquin	5,8	0,2	0,1	74	9,4	49	5,2	51	0,2	–	0,3	–
Leflunomid	6,8	2,8	0,1	0,2	0,3	1,3	3,1	3,0	1,6	0,9	1,6	–
Methotrexat	55	41	7,8	10	25	7,7	43	17	28	31	28	9,1
Mycophenolat	–	0,1	0,1	15	15	0,9	10	3,7	0,0	–	3,6	–
Sulfasalazin	3,4	2,9	3,3	0,0	–	–	–	–	0,2	–	–	0,6

Alle Angaben sind Prozentwerte

*RA* rheumatoide Arthritis, *PsA* Psoriasis-Arthritis, *axSpA* axiale Spondyloarthritis, *SLE* systemischer Lupus erythematodes, *SSc* systemische Sklerose, *SjS* primäres Sjögren-Syndrom, *IIM* idiopathische inflammatorische Myositiden, *MCTD* mixed connective tissue diseases, *NSAR* nichtsteroidale Antirheumatica, *PMR* Polymyalgia rheumatica, *RZA* Riesenzellarteriitis, *AAV* ANCA-assoziierte Vaskulitiden, *BD* Morbus Behçet, *cs* conventional synthetic, *b* biologic, *ts* targeted synthetic, *DMARD* disease-modifying antirheumatic drug

### csDMARDs

Bei RA und SLE hatten 89 % mindestens eine Basistherapie (cs, b oder tsDMARD), bei PsA 85 % und bei AAV 81 %. Methotrexat (MTX) war das am häufigsten verwendete csDMARD. Es wurde vor allem bei RA (55 %), PsA (43 %) und bei idiopathischen inflammatorischen Myositiden (IIM, 43 %) verwendet. Azathioprin kam häufig bei AAV und BD (je 25 %) zum Einsatz. Bei SLE (74 %), MCTD (51 %) und SjS (49 %) war Hydroxychloroquin (HCQ) das häufigste csDMARD. Mycophenolat-Mofetil spielte bei SLE und systemischer Sklerose (SSc, jeweils 15 %) eine Rolle. Leflunomid (6,8 % bei RA), Sulfasalazin, Ciclosporin A und Cyclophosphamid waren nur bei wenigen Patient:innen Bestandteil der Therapie.

### bDMARDs

Biologische (b)DMARDs wurden am häufigsten bei axSpA (63 %) und PsA (50 %) verordnet (s. Tab. 4). Auch bei RZA (36 %), RA (31 %), AAV (30 %) und BD (27 %) erhielt jeder dritte bis vierte Patient ein bDMARD. Häufigste bDMARD-Therapie waren Tumornekrose-Faktor-Inhibitoren (TNFi), vor allem bei axSpA (54 %), PsA (27 %), BD (26 %) und RA (19 %). Bei PsA waren auch IL17-Inhibitoren (16 %), davon häufiger Secukinumab (10,7 %) als Ixekizumab (5,2 %), und IL12/23-Inhibitoren (6 %) relevant.

**Tab. 4:** Biologische und zielgerichtete b/tsDMARDs

	RA	PsA	axSpA	SLE	SSc	SjS	IIM	MCTD	PMR	RZA	AAV	BD
*N mit Therapieangabe*	*5791*	*1715*	*1376*	*890*	*344*	*236*	*97*	*137*	*452*	*339*	*305*	*154*
bDMARD	31	50	63	18	6,2	8,5	13	10	1,3	36	30	27
Abatacept	2,9	0,5	–	0,7	0,3	0,9	–	–	–	–	–	–
Anakinra	0,2	–	–	0,3	–	–	–	0,7	0,2	0,3	0,3	–
Belimumab	–	–	–	15	–	0,4	–	1,5	–	–	–	–
Anifrolumab	–	–	–	0,1	–	–	–	–	–	–	–	–
TNFi	19	27	54	0,3	0,3	0,4	–	–	0,2	1,2	0,7	26
– Adalimumab	7,1	15	23	0,2	0,3	–	–	–	–	0,3	0,7	14
– Certolizumab	2,5	2,5	4,0	–	–	–	–	–	–	–	–	–
– Etanercept	8,1	5,9	12	0,1	–	–	–	–	0,2	0,6	–	–
– Golimumab	1,2	2,2	7,8	–	–	–	–	–	–	–	–	1,9
– Infliximab	0,5	0,9	7,4	–	–	0,4	–	–	–	0,3	–	10
Rituximab	3,5	–	–	1,7	2,1	6,4	13	5,2	–	–	27	0,6
IL-5i: Mepolizumab	–	–	–	–	–	–	–	–	–	–	1,6	–
IL-6Ri	4,3	0,1	0,1	0,3	3,5	0,4	–	2,2	0,9	34	0,3	–
– Tocilizumab	3,3	0,1	0,1	0,2	3,2	0,4	–	2,2	0,9	34	0,3	–
– Sarilumab	1,0	–	–	0,1	0,3	–	–	–	–	–	–	–
IL-12/23i	–	6,3	0,8	0,1	–	–	–	–	–	–	0,3	–
– Guselkumab	–	3,1	0,2	–	–	–	–	–	–	–	–	–
– Risankizumab	–	0,5	–	–	–	–	–	–	–	–	–	–
– Ustekinumab	–	2,8	0,6	0,1	–	–	–	–	–	–	0,3	–
IL-17i	0,2	16	8,2	–	–	–	–	0,7	–	–	–	–
– Ixekizumab	0,1	5,2	0,8	–	–	–	–	–	–	–	–	–
– Secukinumab	0,1	10,7	7,4	–	–	–	–	0,7	–	–	–	–
tsDMARD	11	6,4	2,8	0,7	0,3	0,9	–	3,0	0,4	0,3	0,3	6,5
JAKi	11	3,6	2,7	0,7	0,3	0,9	–	2,2	0,4	0,3	0,3	1,3
– Baricitinib	4,4	0,1	0,1	0,3	0,3	0,4	–	0,7	–	–	0,3	–
– Filgotinib	1,5	–	–	–	–	0,4	–	–	–	–	–	–
– Tofacitinib	1,0	0,8	0,4	0,1	–	–	–	–	–	–	–	1,3
– Upadacitinib	4,3	2,8	2,2	0,2	–	–	–	1,5	0,4	0,3	–	–
Apremilast	–	2,9	0,1	–	–	–	–	0,7	–	–	–	5,2

Alle Angaben sind Prozentwerte

*RA* rheumatoide Arthritis, *PsA* Psoriasis-Arthritis, *axSpA* axiale Spondyloarthritis, *SLE* systemischer Lupus erythematodes, *SSc* systemische Sklerose, *SjS* primäres Sjögren-Syndrom, *IIM* idiopathische inflammatorische Myositiden, *MCTD* mixed connective tissue diseases, *PMR* Polymyalgia rheumatica, *RZA* Riesenzellarteriitis, *AAV* ANCA-assoziierte Vaskulitiden, *BD* Morbus Behçet, *JAKi* Januskinase-Inhibitoren, *IL* Interleukin, *TNFi* Tumornekrosefaktor-Inhibitoren, *bDMARD* biologic DMARD, *tsDMARD* targeted synthetic DMARD, *DMARD* disease-modifying antirheumatic drug

Tocilizumab wurde häufig bei RZA (34 %) eingesetzt, allerdings mit hoher Varianz zwischen den Einrichtungen. Rituximab war die häufigste bDMARD-Therapie bei AAV (27 %), kam aber auch bei RA und off-label bei Kollagenosen zum Einsatz. Belimumab erhielten 15 % der SLE-Patient:innen. Mepolizumab wurde 5 von 51 (9,8 %) der Patient:innen mit einer eosinophilen Granulomatose mit Polyangiitis (EGPA) verordnet.

### tsDMARDs

JAK-Inhibitoren kamen vor allem bei der RA (11 %) zum Einsatz. Es wurden mehrheitlich Baricitinib (4,4 %) und Upadicinib (4,3 %), seltener Filgotinib (1,5 %) und Tofacitinib (1,0 %) verordnet.

Apremilast kam bei BD (5 %) und bei PsA (3 %) zum Einsatz.

### Biosimilars

Der Biosimilar-Anteil unter den TNF-Blockern lag für die RA bei 62 % (Adalimumab), 66 % (Etanercept), 55 % (Infliximab) für die PsA bei 67 % (Adalimumab), 58 % (Etanercept) und 73 % (Infliximab) und für die axSpA bei 67 % (Adalimumab), 53 % (Etanercept) und 72 % (Infliximab). Für Rituximab lag er bei der RA bei 34 % und bei AAV bei 38 %.

### Ergänzende nichtmedikamentöse Therapiemaßnahmen

In den letzten 12 Monaten erhielten 31 % (RA), 33 % (PsA), 22 % (SLE) und 51 % (axSpA) Physiotherapie. Bei schweren Funktionseinschränkungen gemäß FFbH (< 50) lag der Anteil bei 46 % (RA) bzw. gemäß BASFI (> 4) bei 60 % (axSpA). Ergotherapie erhielten 5 % (SLE) bis 8 % (RA). Rheumafunktionstraining wurde von 3–5 % der Betroffenen wahrgenommen.

### Patientenberichtete Outcomes

Die patientenberichtete Krankheitsaktivität lag krankheitsübergreifend im mittleren Bereich (s. Tab. 5). Der Mittelwert nach der NRS lag zwischen 2,8 ± 2,5 (AAV) und 4,2 ± 2,3/2,6 (SSc/IIM). Im Vergleich zur ärztlichen Einschätzung gaben die Betroffenen im Mittel jeweils eine um etwa 2 Einheiten (auf der Skala von 0–10) höhere Krankheitsaktivität an.

**Tab. 5:** Patientenberichtete Outcomes nach numerischen Ratingskalen (0–10)

	RA	PsA	axSpA	SLE	SSc	SjS	IIM	MCTD	PMR	RZA	AAV	BD
Krankheitsaktivität	3,6 (2,4)	3,6 (2,5)	3,8 (2,5)	3,1 (2,4)	4,2 (2,3)	4,1 (2,5)	4,2 (2,6)	3,7 (2,6)	3,8 (2,5)	3,4 (2,4)	2,8 (2,5)	3,4 (2,9)
Gesundheitszustand	4,1 (2,3)	4,1 (2,3)	4,2 (2,3)	3,7 (2,4)	4,6 (2,1)	4,2 (2,2)	4,5 (2,5)	4,3 (2,3)	4,5 (2,3)	4,1 (2,2)	3,9 (2,3)	4,1 (2,7)
Schmerzen	3,7 (2,6)	3,9 (2,6)	4,1 (2,5)	3,2 (2,7)	3,8 (2,6)	3,7 (2,4)	3,8 (2,8)	3,8 (2,9)	3,7 (2,8)	3,2 (2,6)	2,7 (2,5)	3,4 (3,2)
Fatigue	4,1 (2,9)	4,3 (3,0)	4,4 (2,8)	4,2 (3,1)	4,4 (2,9)	4,7 (2,9)	4,2 (3,1)	4,5 (3,2)	3,7 (2,8)	4,1 (2,8)	3,8 (3,0)	4,4 (3,4)
Körperliche Schwierigkeiten	3,6 (2,4)	3,5 (2,4)	3,9 (2,4)	3,1 (2,4)	3,8 (2,4)	3,5 (2,4)	3,9 (2,4)	3,7 (2,4)	3,4 (2,4)	3,5 (2,4)	3,0 (2,4)	3,1 (2,4)
Schlafstörungen	3,9 (3,1)	4,0 (3,2)	4,2 (3,1)	4,2 (3,3)	4,2 (3,1)	4,4 (3,3)	3,7 (3,3)	4,5 (3,2)	3,8 (3,1)	3,9 (3,1)	3,7 (3,1)	4,1 (3,6)
Psychisches Wohlbefinden	3,6 (2,6)	3,6 (2,8)	3,8 (2,6)	3,4 (2,7)	3,9 (2,8)	3,9 (2,7)	3,7 (2,7)	3,9 (2,8)	3,6 (2,7)	3,5 (2,5)	3,3 (2,5)	4,0 (3,2)
Körperliches Wohlbefinden	4,0 (2,5)	4,1 (2,5)	4,2 (2,4)	3,8 (2,6)	4,3 (2,3)	4,4 (2,5)	4,3 (2,5)	4,3 (2,6)	4,0 (2,4)	3,9 (2,4)	3,6 (2,4)	3,9 (3,1)

Alle Angaben werden auf numerischen Ratingskalen von 0–10 erfasst, 0 ist jeweils der beste Wert und entspricht keiner Einschränkung. Angegeben sind jeweils Mittelwert und Standardabweichung

*RA* rheumatoide Arthritis, *PsA* Psoriasis-Arthritis, *axSpA* axiale Spondyloarthritis, *SLE* systemischer Lupus erythematodes, *SSc* systemische Sklerose, *SjS* primäres Sjögren-Syndrom, *IIM* idiopathische inflammatorische Myositiden, *MCTD* mixed connective tissue diseases, *PMR* Polymyalgia rheumatica, *RZA* Riesenzellarteriitis, *AAV* ANCA-assoziierte Vaskulitiden, *BD* Behçet Disease

**Abb. 2:**
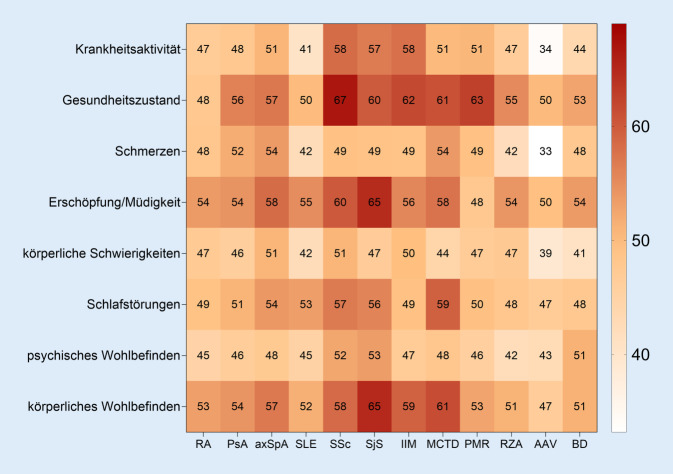
Häufigkeit moderater bis starker Einschränkungen in patientenberichteten Outcomes. Dargestellt ist der prozentuale Anteil an Patient:innen, die für die jeweilige Dimension eine Einschränkung von mindestens 4 bis 10 auf numerischen Ratingskalen angekreuzt haben. *RA* Rheumatoide Arthritis, *PsA* Psoriasis-Arthritis, *axSpA* axiale Spondyloarthritis, *SLE* systemischer Lupus erythematodes, *SSc* systemische Sklerose, *SjS* primäres Sjögren Syndrom, *IIM* idiopathische inflammatorische Myositiden, *MCTD* mixed connective tissue diseases, *PMR* Polymyalgia rheumatica, *RZA* Riesenzellarteriitis, *AAV* ANCA-assoziierte Vaskulitiden, *BD* Behcet Disease

Am häufigsten stuften Patient:innen mit Kollagenosen ihre Krankheitsaktivität als moderat bis hoch (NRS 4–10) ein (s. Abb. 2). Mehr als die Hälfte aller Patient:innen mit Kollagenosen beurteilten auch ihren allgemeinen Gesundheitszustand, das körperliche Wohlbefinden und Erschöpfung/Müdigkeit als moderat bis stark eingeschränkt bzw. hoch. Bis auf AAV- und RZA-Betroffene dokumentierten Patient:innen diagnoseübergreifend zu etwa 50 % moderate bis starke Schmerzen.

### Weitere versorgungsrelevante Daten

Von den Betroffenen hatten im Vorjahr 8 bis 10 % aufgrund ihrer rheumatischen Erkrankung (RA, axSpA, SLE, PsA) einen stationären Aufenthalt mit einer Dauer von 8 Tagen (RA, PsA) bis 14 Tagen (SLE) im Median. Eine ambulante oder stationäre medizinische Rehabilitationsmaßnahme wurde bei 7,2 % (RA), 6,4 % (PsA), 9,2 % (axSpA) und 6,7 % (SLE) der Patient:innen durchgeführt. Mitglied einer Patientenorganisation waren 9,5 % (RA), 6,6 % (PsA), 10 % (axSpA) bzw. 14 % (SLE).

Der Anfahrtsweg zur rheumatologischen Einrichtung betrug für 28 % der Patient:innen 0–10 km, für 26 % 11–20 km, für 33 % 21–50 km und für 14 % > 50 km. Die mittlere Entfernung zum Wohnort betrug 23 km in Großstädten/Metropolen, 31 km in Mittelstädten, 51 km in der Kleinstadt und 50 km auf dem Land.

## Diskussion

Mithilfe der Kerndokumentation können aktuelle Daten zur Versorgung von Patient:innen mit entzündlich-rheumatischen Erkrankungen aus derzeit 13 rheumatologischen Einrichtungen berichtet werden. Im Jahr 2022 zeigte sich im Vergleich zu den Vorjahren krankheitsübergreifend ein weiterer Rückgang im Einsatz von GC [1]. Bei RA blieb die Häufigkeit einer bDMARD-Therapie mit 31 % zum Vorjahr unverändert, auch tsDMARDs wurden mit 11 % nur gering häufiger verordnet als im Vorjahr (10 %) [1]. Bei PsA stieg der Einsatz von IL-17- und IL-12/3-Inhibitoren, aber TNFi blieben mit 27 % das häufigste bDMARD für die Behandlung der PsA. Die axSpA-Therapie veränderte sich im Vergleich zu den Vorjahren kaum. Jede zweite axSpA-Patient:in erhielt einen TNF-Inhibitor. Von den mit einem bDMARD behandelten axSpA-Patient:innen hatten 53 % zusätzlich ein NSAR, 30 % kontinuierlich und 70 % bedarfsorientiert. Mit einer NSAR-Monotherapie wurden 23 % behandelt.

Drei von vier SLE-Patient:innen erhielten HCQ, der Anteil ist gestiegen. Auch Belimumab wurde im Vergleich zum Vorjahr (11 % vs. 15 %) häufiger verwendet [1]. Die Behandlung von SjS, SSc und IIM blieb heterogen, mit Rituximab und Tocilizumab als bDMARD-Optionen. Bei den Vaskulitiden kam bei RZA zunehmend Tocilizumab zum Einsatz. Bei EGPA wurden 2022 die ersten Patient:innen mit Mepolizumab behandelt. Eine Übersicht über die Medikation der letzten 15 Jahre wird jedes Jahr in Ergebnisgrafiken dargestellt [1].

Die überwiegende Zahl an Patient:innen aus der Kerndokumentation hat eine lange Krankheitsdauer. Während die Krankheitsaktivität bei diesen langjährig Erkrankten ärztlicherseits größtenteils als niedrig eingestuft wurde, dokumentierten viele Betroffene weiterhin moderate bis starke Einschränkungen hinsichtlich Schmerzen, Beweglichkeit, Müdigkeit/Erschöpfung, der Schlafruhe und in ihrem Wohlbefinden. Die Diskrepanz zwischen ärztlich- und patientenberichteter Krankheitsaktivität ist in zahlreichen Studien beschrieben und wird darauf zurückgeführt, dass Ärzt:innen und Betroffene unterschiedliche Faktoren (u. a. laborchemische Entzündungsaktivität, Organbeteiligung vs. Fatigue, Schmerz, Coping, eingeschränkte Teilhabe) bei ihrer Beurteilung aktiver Krankheitsmanifestationen berücksichtigen [4–6]. Die patientenberichteten Einschränkungen scheinen auf die vielfältigen medikamentösen Behandlungsoptionen noch nicht in ausreichendem Maße anzusprechen oder haben sich bereits chronifiziert.

### Limitationen und Stärken

Die Teilnahme an der Kerndokumentation ist freiwillig und erfordert von Rheumatolog:innen und Betroffenen eine hohe Bereitschaft, jedes Jahr viele Daten zu dokumentieren. Die Dokumentation wird in begrenztem Umfang vergütet. Die Einrichtungen sind bundesweit vertreten und umfassen mit Einzelpraxen, Gemeinschaftspraxen, Kliniken und Hochschulambulanzen alle ambulanten Versorgungssektoren der Rheumatologie. Mit knapp 14.000 Patient:innen bildet die Kerndokumentation etwas weniger als 1 % der geschätzten Zahl Betroffener in Deutschland (etwa 1,8 Mio. [7]) ab. Nicht erfasst werden Betroffene, die nicht fachärztlich versorgt werden. Die Daten sind daher nicht repräsentativ für die Versorgung aller Betroffener in Deutschland, liefern aber eine multizentrische und sektorenübergreifende Abbildung der fachärztlichen rheumatologischen Versorgung. Die kontinuierliche Erfassung über viele Jahre ermöglicht die Darstellung von Versorgungstrends in Therapie und patientenberichteten Outcomes.

## Fazit

Der Jahresbericht aus der Kerndokumentation zeigt aktuelle medikamentöse und patientenberichtete Versorgungsdaten aus Deutschland zu den häufigsten entzündlich-rheumatischen Erkrankungen. Die beobachteten Veränderungen sind im Einklang mit entsprechenden Empfehlungen. Die vergleichende Übersicht zu den einzelnen Diagnosen ergänzt die jährlich erscheinenden Ergebnisgrafiken und soll zukünftig einmal im Jahr berichtet werden.
